# The Mutational Landscape of Myeloid Leukaemia in Down Syndrome

**DOI:** 10.3390/cancers13164144

**Published:** 2021-08-18

**Authors:** Carini Picardi Morais de Castro, Maria Cadefau, Sergi Cuartero

**Affiliations:** 1 Josep Carreras Leukaemia Research Institute (IJC), Campus Can Ruti, 08916 Badalona, Spain; cpicardi@carrerasresearch.org (C.P.M.d.C); mcadefau@carrerasresearch.org (M.C.); 2 Germans Trias i Pujol Research Institute (IGTP), Campus Can Ruti, 08916 Badalona, Spain

**Keywords:** myeloid leukaemia, Down syndrome, trisomy 21, acute megakaryoblastic leukaemia (AMKL), transcription, chromatin, signalling

## Abstract

**Simple Summary:**

Leukaemia occurs when specific mutations promote aberrant transcriptional and proliferation programs, which drive uncontrolled cell division and inhibit the cell’s capacity to differentiate. In this review, we summarize the most frequent genetic lesions found in myeloid leukaemia of Down syndrome, a rare paediatric leukaemia specific to individuals with trisomy 21. The evolution of this disease follows a well-defined sequence of events and represents a unique model to understand how the ordered acquisition of mutations drives malignancy.

**Abstract:**

Children with Down syndrome (DS) are particularly prone to haematopoietic disorders. Paediatric myeloid malignancies in DS occur at an unusually high frequency and generally follow a well-defined stepwise clinical evolution. First, the acquisition of mutations in the GATA1 transcription factor gives rise to a transient myeloproliferative disorder (TMD) in DS newborns. While this condition spontaneously resolves in most cases, some clones can acquire additional mutations, which trigger myeloid leukaemia of Down syndrome (ML-DS). These secondary mutations are predominantly found in chromatin and epigenetic regulators—such as cohesin, *CTCF* or *EZH2*—and in signalling mediators of the JAK/STAT and RAS pathways. Most of them are also found in non-DS myeloid malignancies, albeit at extremely different frequencies. Intriguingly, mutations in proteins involved in the three-dimensional organization of the genome are found in nearly 50% of cases. How the resulting mutant proteins cooperate with trisomy 21 and mutant GATA1 to promote ML-DS is not fully understood. In this review, we summarize and discuss current knowledge about the sequential acquisition of genomic alterations in ML-DS.

## 1. Introduction

Trisomy 21 is the most common chromosomal disorder in humans and is the genetic basis of Down syndrome (DS) [1]. This multisystem disorder results in numerous phenotypic features, including craniofacial abnormalities and cognitive impairment [2]. Although solid tumours are less frequent in the DS than the non-DS population [3,4,5], individuals with DS have a higher risk of developing haematopoietic disorders [4]. DS neonates have a variety of haematological abnormalities such as high haemoglobin concentration, large mean cell volume, erythroblastosis, high leukocyte count and low frequency of platelets [6]. DS children are at greater risk of developing acute lymphoblastic leukaemia (ALL) and acute myeloid leukaemia (AML), by an estimated factor of 27-fold and 150-fold compared with the general population, respectively [4,7]. AML is a heterogeneous group of myeloid leukaemias that originate from clones of haematopoietic stem and progenitor cells (HSPCs) and myeloid lineage precursors carrying genetic mutations that alter cell proliferation and compromise differentiation. In myeloid leukaemia of Down syndrome (ML-DS), most cases phenotypically reflect acute megakaryoblastic leukaemia (AMKL), a rare subtype of AML in which mutations compromise megakaryocytic maturation.

**Figure 1:**
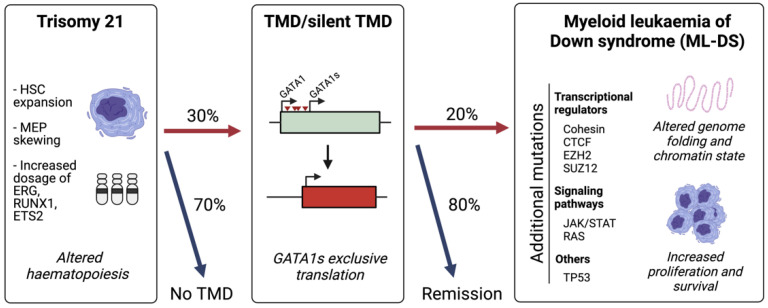
The sequential acquisition of mutations in ML-DS. Trisomy 21 alters foetal haematopoiesis causing an expansion of haematopoietic stem cells (HSCs) with skewing towards megakaryocyte-erythroid progenitors (MEPs). A total of 30% of Down syndrome (DS) neonates acquire mutations in *GATA1* which lead to the exclusive production of the short isoform, GATA1s, which promotes a transient myeloproliferative disorder (TMD). A total of 20% of children with TMD will develop myeloid leukaemia of Down syndrome (ML-DS) following the acquisition of secondary mutations.

ML-DS is characterized by a distinctive multi-step evolution in which it is always preceded by a pre-leukaemic condition known as transient abnormal myelopoiesis (TAM) or transient myeloproliferative disorder (TMD) [6,8] (Figure 1). All TMD cases harbour mutations in the haematopoietic transcription factor GATA1 [9,10]. The disorder occurs in approximately 10% of DS newborns and is usually diagnosed 2 months after birth by the identification of a high number of immature blasts in the circulating blood [8,11,12]. However, the presence of GATA1 mutations in up to 30% of DS newborns indicates an additional 20% of undetected or silent TMD cases [13]. Children with TMD may develop severe symptoms such as thrombocytopaenia, leukocytosis, anaemia, lymphocytosis and liver failure, and approximately 20% of patients do not survive [13,14,15,16,17]. In the majority of TMD and silent TMD cases, the patient goes into permanent remission without any treatment. However, 20–30% of children with TMD develop ML-DS before 5 years of age [18,19,20]. ML-DS is characterized by a low number of white blood cells and high concentrations of immature blasts and dysplastic myeloid cells. Around 80% of children with ML-DS respond well to chemotherapy and survive, but the other 20% suffers from relapse [8,17], highlighting the need of new therapeutic approaches.

The molecular mechanisms involved in the progression from TMD to ML-DS are not fully understood. Large-scale sequencing studies have identified the most frequent mutations acquired in ML-DS in addition to *GATA1* mutations [9,10,21]. Strikingly, approximately half of the cases have mutations in cohesin or *CTCF*, the two main drivers of three-dimensional (3D) genome folding. This high frequency is unexpected, especially for *CTCF* which is rarely mutated in AML or other myeloid malignancies. Moreover, chromatin modifiers such as *EZH2* are more frequently mutated in ML-DS than in AML. Signalling pathway mutations are mostly found in receptors and members of the Janus kinase-signal transduction and activator of the transcription (JAK-STAT) signalling cascade, as well as in the RAS pathway. These mutations may interfere or cooperate with altered signalling pathways in DS. In general, however, the interplay between the three genetic elements comprising this disease—trisomy 21, *GATA1* mutations and secondary mutations—is poorly understood. Here, we provide an overview of the current knowledge on how these elements may promote ML-DS.

## 2. Altered Haematopoiesis in Down Syndrome

Trisomy 21 foetal livers have an abnormal haematopoietic development. Specifically, there is an increased frequency of haematopoietic stem cells (HSCs), which also show increased clonogenicity and megakaryocytic–erythroid output. This results in an expansion of megakaryocyte–erythroid progenitors (MEPs) and a decrease in granulocyte–macrophage progenitors (GMP). In addition, B-cell differentiation is impaired [22,23,24,25,26,27]. The mechanisms by which the extra copy of chromosome 21 alters normal blood production remain unclear. Several genes with important functions in haematopoietic development, including *ERG*, *ETS2* and *RUNX1,* are located on chromosome 21. Overexpression of *Erg* and *Ets2* induce megakaryocytic expansion and contribute to a myeloproliferative phenotype in mice [28,29,30]. In addition, overexpression of another chromosome 21 gene, *Dyrk1a*, induces a marked megakaryocytic expansion [31]. However, the role of the increased dosage of chromosome 21 genes on altered trisomic foetal liver haematopoiesis is unclear. On one hand, foetal liver HSCs only show extremely modest increases in expression of *ERG* and *RUNX1* [26]. On the other, targeted deletion of *RUNX1*, *ETS2* and *ERG* in human trisomy 21-induced pluripotent stem cells (iPSCs) suppresses the altered differentiation phenotypes [24].

Trisomy 21 can also interfere with gene expression by altering DNA methylation patterns. DNA methylation profiling at different stages of ML-DS development revealed hypomethylation in early stages and hypermethylation in advanced stages [32]. Interestingly, loss of methylation was found to affect genes associated with developmental disorders, while gain of methylation was observed in key genes involved in haematopoiesis. Similarly, haematopoietic cells of DS newborns have differential methylation patterns at promoter/enhancer regions relative to non-DS newborns [33]. Specifically, the promoter regions of two genes involved in megakaryopoiesis (*RUNX1* and *FLI1*) were found to be hypermethylated in DS samples [33].

Different mouse models of partial trisomy 21 have been used to understand DS haematopoietic development. In mice, the orthologous regions of human chromosome 21 are located in chromosomes 10, 16 and 17. The most commonly used DS mouse model (Ts65Dn) carries an extra copy of 104 genes of mouse chromosome 16 [34]. These mice display megakaryocytic hyperplasia, thrombocytosis and myelofibrosis in adults [35]. Narrowing down the number of trisomic genes required to develop these phenotypes, the Ts1Rhr strain only carries 33 orthologous genes in the human Down syndrome critical region (DSCR) and adult mice show an altered haematopoietic phenotype, including progressive thrombocytosis, increased number of megakaryocytes and altered proportion of myeloid progenitors. This strain has been used to model ML-DS by adding a second genetic event (a *Gata1* mutation) and a third event (*Mpl*^W515L^) [31]. While these strains may not recapitulate all aspects of DS—especially in the foetal liver—their phenotype supports a role of at least some of the genes in human chromosome 21 in maintaining a correct blood differentiation balance, and can be used as a model to identify the mechanisms driving leukaemia in DS [29,31].

## 3. Mutations in *GATA1* Cause a Transient Myeloproliferative Disorder

The GATA family consists of six transcription factors (GATA1 to GATA6) that bind to the same DNA consensus sequence through a highly conserved zinc finger domain [36,37]. According to their expression patterns, GATA transcription factors can be divided into two subfamilies. *GATA1*, *GATA2* and *GATA3* are expressed in haematopoietic cells, while *GATA4*, *GATA5* and *GATA6* are expressed in various tissues including the heart, intestine and lung [38]. In humans, *GATA1* is located on chromosome X [39] and is mainly expressed in erythrocytes [40,41], eosinophils [42], mast cells [43], megakaryocytes [43,44] and Sertoli cells [45,46]. The gene has six exons, and it can be translated into two distinct isoforms: the full-length isoform and a short isoform, termed GATA1s, which excludes exon 2. This results in a shorter protein that lacks 83 amino acids of the N-terminal region of the long isoform [47]. GATA1 has an essential role in the determination of the erythroid and megakaryocytic lineages in haematopoiesis. Conditional deletion of *Gata1* in mice leads to impaired erythropoiesis. In addition, mice with selective loss of *Gata1* expression in the megakaryocytic lineage have a markedly lower frequency of platelets and impaired megakaryopoiesis [48].

All TMD and ML-DS cases carry somatic mutations in *GATA1* resulting in the introduction of a premature stop codon which promotes the exclusive translation of GATA1s [49]. Mechanistically, the long and short GATA1 isoforms have similar but non-identical binding patterns on the genome [27,50,51,52]. MEP-specific genes and enhancers are differently bound by the two isoforms, which could explain the skewed differentiation profile of GATA1s-expressing cells. The short isoform is less efficient at activating erythroid gene pathways in MEPs than the full-length GATA1 [50,52]. Moreover, analysis of chromatin occupancy and gene expression during erythropoiesis of GATA1s mice shows that normal murine foetal haematopoiesis is impaired as a result of deregulation in gene pathways of erythroid and megakaryocytic lineages. Interestingly, while GATA1s binding is reduced at erythroid genes, its activity is enhanced at important megakaryocytic genes [51,52].

Cellular models of TMD and GATA1s show that trisomy 21 and *GATA1* mutations lead to aberrant and accelerated production of poorly differentiated haematopoietic cells [24]. During haematopoiesis, GATA1s increases the fraction of myeloid and megakaryocyte progenitors [53]. Specifically, immature kit-expressing CD41^hi^ megakaryocyte precursors accumulate during late megakaryopoiesis, causing a reduction in apoptosis and an increase in the number of cells in S-phase [54]. Moreover, GATA1s is upregulated by trisomy 21 [24], favouring the accumulation of immature blasts in the circulating blood, which in turn, are susceptible to acquired additional mutations and progression to ML-DS.

## 4. Spectrum of Mutations Driving the Transition from TMD to ML-DS

The progression from TMD to ML-DS requires at least one additional mutation in the GATA1s clones. The advent of large-scale sequencing studies over the last decade has enabled the identification of recurrent mutations in ML-DS patients [9,10,21]. The most frequent alterations can be grouped into two major categories: genes encoding for transcriptional regulators and genes encoding for signalling pathway mediators. Strikingly, among the first group, the most frequently mutated genes belong to the cohesin complex. Between 38% and 53% of patients have mutations in cohesin, compared to only 11% of non-DS AMKL patients [9,21]. This may indicate that the selective advantage of cohesin mutations in ML-DS could be related to trisomy 21-specific features. CTCF, which cooperates with cohesin in the formation of topologically associating domains (TADs), is also extremely frequently mutated (11–20% of cases). In contrast to cohesin, *CTCF* is also recurrently mutated in non-DS AMKL (10–21%) [9,55]. In addition, mutations in epigenetic regulators are also frequent, such as in the polycomb repressive complex 2 (PRC2) components *EZH2* and *SUZ12* or the chromatin modifier *KANSL1*. Analysis of the clonal origin of mutations shows that cohesin, *CTCF* and *EZH2* mutations may have essential roles in the early stages of ML-DS progression [9]. Deletions, missense, nonsense and frameshift mutations, usually leading to a loss of function, are found in transcription factors including *RUNX1*, *TP53*, *WT1*, *CREBBP* and *MYC* (~8%). The spliceosome component *SRSF2* is mutated in ~8.5% of cases. Among signalling-related genes, the JAK-STAT pathway shows the higher frequency of mutations (48%), followed by members of the RAS family (14%) [21]. Despite this detailed knowledge on the identity and frequency of the recurrently mutated genes in ML-DS, further studies are needed to assess their prognostic value.

**Figure 2:**
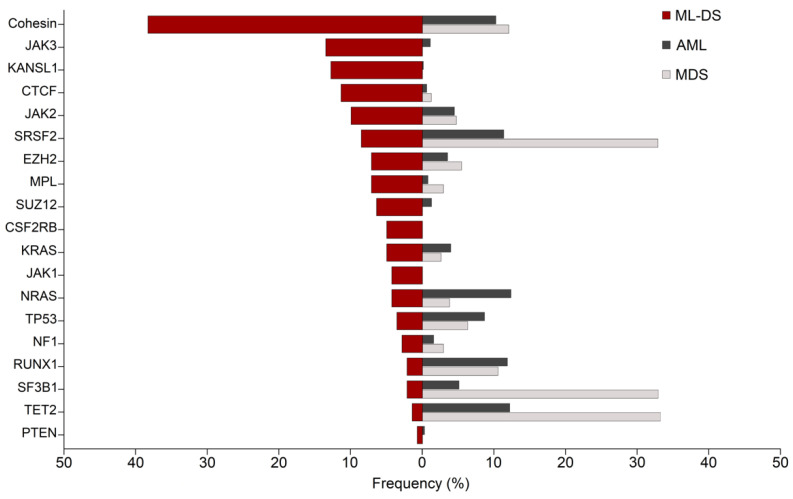
Comparative frequencies of commonly mutated genes in ML-DS, AML and MDS. Red bars show the frequencies of patients harbouring the most common mutations in myeloid leukaemia of Down syndrome (ML-DS). Dark and light grey bars show their frequency in acute myeloid leukaemia (AML) and myelodysplastic syndromes (MDS), respectively [21,57,62]. Cohesin mutations represent the cumulative frequencies of the cohesin subunits *STAG2*, *RAD21*, *SMC1A*, *SMC3* and *NIPBL*. Figure inspired by [63].

Most of these mutations are also found in AML and other myeloid malignancies, but the frequencies at which they are found can be extremely different (Figure 2). Cohesin is mutated in 10–12% of cases in AML, and *CTCF* in less than 1%. *JAK1* and *JAK3* are rarely mutated in AML, whereas *NRAS* is more often found mutated in AML than in ML-DS. In addition, the most frequent mutations in AML are almost absent in ML-DS. This is the case of *FLT3*, *DNMT3A* and *NPM1* [56,57], and of other recurrent mutations such as *IDH1/2* or *CEBPα*. Similarly, spliceosome and epigenetic proteins such as SF3B1 and TET2, which are found among the most frequently mutated proteins in myelodysplastic syndromes (MDS), are only mutated in less than 3% of cases in ML-DS.

While ML-DS and non-DS AMKL share common morphological and immunophenotypic features [58,59], they have different genetic backgrounds. The most remarkable difference is that non-DS AMKL patients usually have fusion events [55,60], such as fusions involving HOX proteins or other haematopoietic factors such as BCR-ABL1, MAP2K2-AF10 and MN1-FLI1 [55,61]. Interestingly, gains of chromosome 21 and *GATA1* mutations occur in 39.2% and ~10.0% of cases, respectively, even though none exhibit physical phenotypes consistent with DS. Paired-sample analyses reveal that 90% of patients harbouring *GATA1* mutations also carry an extra copy of chromosome 21 [55].

### 4.1. Mutations in Transcriptional Regulators and Chromatin Modifiers

Transcriptional and chromatin regulators are extremely frequently mutated in myeloid malignancies [57,64], including ML-DS. However, while the most frequently mutated proteins in clonal haematopoiesis of indeterminate potential (CHIP), MDS and AML include regulators of DNA methylation or spliceosome components, the highest frequencies in ML-DS are found in cohesin subunits and CTCF (Figure 2). In addition, ~30–40% carry mutations in the PRC2 members EZH2 and SUZ12 and other epigenetic modifiers [9,21]. Here, we summarize current knowledge about the roles of these factors in leukaemia.

#### 4.1.1. Mutations in the Cohesin Complex

Mutations in members of the cohesin complex, including *STAG2*, *SMC1A*, *SMC3*, *RAD21* and *NIPBL*, are frequently found in several cancer types such as bladder cancer, Ewing sarcoma and glioblastoma [65]. Cohesin is also recurrently mutated in AML (12–18% of cases) and in other myeloid malignancies, such as MDS and chronic myelomonocytic leukaemia (CMML) [57,66,67,68]. Due to cohesin’s essential role in chromosome segregation during mitosis, it was initially thought that the main tumorigenic effect of cohesin mutations was to promote genome instability. However, several studies disproved this hypothesis as cohesin-mutant cells do not display greater levels of aneuploidy than non-cohesin-mutant cells [69,70,71]. Mutations in the different subunits occur in a mutually exclusive manner [9,57,68]. Consistent with the requirement for a minimal dose of functional cohesin for cell proliferation [72], mutations are always heterozygous. This is compatible with cell-cycle progression and explains the absence of increased rates of aneuploidy. As in most solid tumours, the most frequently mutated cohesin subunit in ML-DS is *STAG2*, representing ~42% of all cohesin mutations [9,21]. These lesions can be missense, nonsense, splice-site changes or large deletions, leading to a predicted loss of function [9,21]. The prognostic impact of cohesin mutations in myeloid malignancies is unclear, since separated studies have reported positive [73], negative [66] and non-significant [67] associations with overall survival.

In order to understand the function of the cohesin complex in normal and malignant haematopoiesis, several groups have studied the impact of partially or totally deleted cohesin in haematopoietic cells [69,71,74,75,76,77,78]. Mouse models of *Smc3* or *Stag2* conditional haploinsufficiency [69,74], as well as those expressing shRNAs targeting different cohesin subunits [71], all display alterations in the cellular composition of the haematopoietic progenitor compartments of the bone marrow. When plated in cytokine-supplemented methylcellulose, cohesin-deficient HSPCs show a greater self-renewal capacity compared with wild-type cells. Together, these results indicate that cohesin is required to maintain a normal balance between self-renewal and differentiation of mouse HSPCs. However, despite displaying features of myeloid dysplasia, cohesin-deficient mice do not develop AML unless they harbour a cooperating mutation [69]. In line with these results, cohesin deficiency or over-expression of the mutant proteins in human HSPCs also leads to an increased self-renewal capacity and impaired differentiation [77,78]. Importantly, a CRISPR knock-out screen in mice with a disomic genetic background did not show increased expansion of cohesin knock-out cells, suggesting that trisomy 21 may increase their leukaemogenic potential [21]. On the other hand, CRISPR/Cas9 editing in trisomy 21 foetal liver progenitors indicates that, while trisomy 21 is required for pre-leukemic initiation by GATA1s, it is dispensable for leukemic progression upon *STAG2* knock-out [27].

Apart from its role in sister chromatid cohesion, cohesin has a critical function in the 3D organization of the genome. The formation of TADs is thought to result from the combined action of cohesin and the DNA-binding factor CTCF. Current models posit that cohesin extrudes chromatin until it encounters two convergently oriented CTCF molecules, resulting in chromatin loops anchored at CTCF sites [79,80,81]. As a consequence, the region located within the two CTCF sites is constantly brought into close proximity, which promotes the physical interactions between genes and regulatory elements [82,83]. Surprisingly, despite this genome-wide role in TAD formation, the complete ablation of cohesin or CTCF does not alter transcription of the great majority of expressed genes [76,84,85,86,87]. However, cohesin depletion does alter the expression of genes whose expression is highly dependent on enhancer elements, such as developmental [82] or inducible [76,88] genes. One example is the requirement for cohesin by macrophages and HSPCs to mount an effective inflammatory response [76,88]. In this case, the absence of cohesin results in weaker interactions between enhancers and promoters of key upstream inflammatory regulators, such as interferon receptor genes or pro-inflammatory transcription factors.

The deregulation of specific subsets of enhancer-dependent genes may explain the altered balance of HSPC subsets and impaired lineage commitment of cohesin-deficient mouse models. Inflammatory signals are key regulators of the balance between self-renewal and differentiation of HSPCs [89,90,91]. The decreased inflammatory responsiveness of cohesin-deficient HSPCs can alter this balance and impair their differentiation capacity [76]. Another example of cohesin-dependent enhancer–promoter contacts is in the *Ebf1* gene, a key lymphoid lineage determining transcription factor. In conditional mouse models of *Stag2* deficiency, the altered 3D structure surrounding *Ebf1* impairs its expression, leading to a blockade of B-cell differentiation [74]. It has also been noted that cohesin mutations frequently co-occur with *RUNX1* mutations in MDS. STAG2 and RUNX1 colocalise at a subset of enhancer elements, and the combined absence of the two factors alters chromatin contacts in a cooperative manner [92]. An interplay between cohesin and specific haematopoietic transcription factors has also been observed in erythroid differentiation, where cohesin displaces *Etv6* from its binding sites, which impairs normal differentiation [93].

Different therapeutic strategies have been proposed to target cohesin-mutant cells (reviewed in [94]). Recently, it has been shown that cohesin-mutant cells are more sensitive to PARP inhibition than cohesin wild-type cells, which could be used therapeutically to treat cohesin-mutant AML [95]. Another recent study has found that an agonist of Wnt signalling specifically inhibits the growth of cohesin-deficient cells [96]. Importantly, the authors showed that cohesin depletion in the ML-DS cell line CMK rendered these cells especially sensitive to Wnt signalling. This finding suggests that Wnt agonists could potentially become an effective therapeutic strategy to treat cohesin-mutant ML-DS.

#### 4.1.2. Mutations in CTCF

*CTCF* mutations are recurrently observed in both ML-DS (11.3–20.0%) and non-DS AMKL (10–21%). These can be frameshift, nonsense, missense or splice-site mutations, and they mostly occur in the region containing the zinc finger domains [9,21,55], where presumably they impair DNA binding [97,98]. A total of 30% of *CTCF* mutations are due to large deletions [9,21]. The observed variant allele frequency (VAF) is similar to that of *GATA1*, suggesting that they are early events in the clonal expansion during the TMD to ML-DS transition [9]. Despite their high frequency in ML-DS, *CTCF* mutations are much less frequent in AML (2%) [99] and in MDS (~1.3%) [62]. In contrast, they are recurrently found in lymphoid leukaemia, including B-cell ALL (0.3–4.2%) [100,101] and T-cell ALL (4.5–6.45%) [101,102,103].

As part of its key role in the formation of TADs [81,104,105,106,107], CTCF has been implicated in the control of gene expression during haematopoietic development [108,109,110,111]. Specifically, CTCF is required for the normal proliferation and differentiation of erythroblasts [108,109,110]. Cell type-specific CTCF binding sites that are acquired during erythroblastic differentiation are enriched in lineage-determining transcription factors such as GATA1 and TAL1. These newly acquired CTCF sites are associated with genes involved in primitive erythrocyte differentiation, suggesting that CTCF dynamic binding might be required for the expression of erythroblastic differentiation genes [109]. The cell-type specificity in CTCF binding is also observed in AML cells. Blasts show enriched CTCF binding at enhancers compared to normal bone marrow cells. In addition, AML specific CTCF binding sites are correlated with cell fate genes and are enriched in key myeloid transcription factors involved in AML pathogenesis, including CEBPA, ETS1, PU.1 and RUNX1 [112]. Whether this aberrant CTCF binding pattern is a cause or a consequence of the altered transcriptional program of AML blasts is not known.

*Ctcf*-hemizygous mice features altered CTCF binding at poorly conserved sites. Consequently, several hundred genes are deregulated, many of which are involved in cancer signalling pathways such as PI3K-AKT [113]. The loss of one *Ctcf* allele in mice results in more aggressive tumours, with high rates of local invasion and metastatic dissemination [114]. Additionally, when *CTCF* expression is induced in cancer cell lines, it promotes a decrease in proliferation and in clonogenic capacity [115]. The opposite association occurs in mouse embryonic fibroblasts, whereby *Ctcf* haploinsufficiency promotes cellular proliferation, colony formation and cell cycle progression [116]. It is therefore not surprising that mutations in *CTCF* are found in many cancers, including uterine (~25%), stomach (~7%), bladder (~6%) and breast-invasive carcinoma (~4%) [114,117,118,119,120,121,122].

As in the case of cohesin mutations, the unusually high frequency found in ML-DS suggests that the partial absence of CTCF function may cooperate with the dosage imbalance of chromosome 21 genes, with specific megakaryocytic transcriptional regulators, or with both. Unlike cohesin knock-out, however, *CTCF* knock-out trisomic GATA1s progenitors from foetal liver did not drive leukemic transformation upon transplantation in mice [27], suggesting that cohesin and *CTCF* mutations may not promote the same leukaemogenic effects, and that *CTCF* mutation may require additional events on top of *GATA1* mutations. Further studies set in a DS genetic background are needed to elucidate the role of CTCF in this disease and to identify actionable targets.

#### 4.1.3. Mutations in PRC2 Members

EZH2 and SUZ12 are subunits of the PRC2 complex that catalyses the deposition of di- and tri-methylation on H3K27, which is then recognized by PRC1, which mono-ubiquitinates H3K119. Altogether, the activity of PRC1 and PRC2 promotes chromatin compaction and transcriptional silencing [123]. Both genes are frequently mutated in ML-DS (7% and 6% of cases, respectively) [21]. In many cancers, *EZH2* is over-expressed [124,125] or carries gain-of-function mutations, such as in germinal center-like diffuse large B-cell lymphoma or follicular B-cell lymphoma [126]. However, myeloid malignancies often display recurrent inactivating mutations, including MDS/MPN (10–13%), myelofibrosis (13%), MDS (5%) and, more rarely, AML [57,62,127,128]. It is therefore not unexpected to find inactivating mutations or deletions of the *EZH2* gene in ML-DS [21]. However, the frequency of these lesions is significantly higher than in general AML (3.54%) [56] (Figure 2). *EZH2* mutations in MDS and AML are associated with bad prognosis and acquired chemoresistance [129,130]. Due to the large number of genes potentially affected by EZH2 inactivation, it has been difficult to pinpoint a specific mechanism of leukaemogenesis. Conditional *Ezh2* or *Suz12* loss-of-function mouse models show increased HSC repopulation capacity, suggesting that normal levels of PRC2 restrict HSC activity [131]. EZH2 inactivation promotes *HOX* genes de-repression, which can be reversed by a combination of bortezomib and cytarabine [130]. It has been shown that *EZH2* mutations can cooperate with other mutations to promote malignancy, such as with *RUNX1* [132] and *NRAS* [133]. This might also be the case in ML-DS, since the *RUNX1* gene resides on chromosome 21 and *NRAS* mutations are frequently found in the disease. Similar to the case of *CTCF*, *EZH2* knock-out foetal liver progenitors with trisomy 21 and GATA1s expression did not drive leukemic transformation upon transplantation in mice [27]. This supports the hypothesis that *EZH2* mutations act in cooperation with additional mutations in ML-DS.

### 4.2. Altered Signalling Pathways in ML-DS

Due to the increased dosage of certain genes located on chromosome 21, DS individuals have constitutive alterations in specific signalling pathways. Examples are genes of the interferon pathway, including *IFNAR1*, *IFNAR2*, *IFNGR2* and *IL10RB*, which reside on chromosome 21. The result of their increased expression is that not only the pathway is aberrantly activated at baseline, but also shows an abnormally elevated response to interferon stimulation [134]. The intensity of interferon activation substantially increases from foetal to adult haematopoiesis. As interferon has an anti-proliferative effect, this has been proposed to contribute to the resolution of TMD at the transition from foetal liver to bone marrow haematopoiesis [135]. DYRK1A and DSCR1 are two proteins encoded by chromosome 21 genes and which negatively regulate the calcineurin/NFAT pathway. Their increased dosage in DS may be responsible for altered megakaryopoiesis through suppression of calcineurin/NFAT [31,136]. In addition, ML-DS cells show hyperactive IGF signalling which cooperates with GATA1s to increase proliferation of blasts [137]. In addition to these trisomy 21-intrinsic alterations in signalling pathways, in ML-DS different members of the JAK-STAT and RAS pathways are recurrently mutated.

#### 4.2.1. Mutations in the JAK-STAT Pathway

The JAK-STAT signalling pathway is critical for haematopoietic development and immune function as it is involved in the rapid transduction of multiple cytokines, hormones and growth factor signals from the cell membrane to the nucleus. Specifically, the JAK-STAT pathway mediates the transmission of type I and type II cytokines, including interleukins, interferons, erythropoietin (EPO), thrombopoietin (TPO), GM-CSF, prolactin and the growth hormone [138]. The binding to their cognate transmembrane receptor leads the receptor to oligomerize, followed by transactivation of JAK tyrosine kinases and phosphorylation of the cytoplasmic tails of the receptors. There are four JAK tyrosine kinases (*JAK1*, *JAK2, JAK3* and *TYK2*), all of which are ubiquitously expressed, except for *JAK3*, which is restricted to a few tissues, including the haematopoietic system [138,139]. Members of the STAT family of proteins bind the phosphorylated receptors, which leads to them being phosphorylated by JAK kinases. This triggers their release from the receptor, oligomerization and nuclear translocation [140]. Once in the nucleus, STAT oligomers bind gene promoters but mostly localize at enhancers, where they distally modulate gene transcription [141]. Unphosphorylated STATs (uSTATs) also have a gene regulatory role. uSTAT5 acts as a negative regulator of megakaryocytic differentiation by competing with ERG to bind near CTCF sites. Activation of STAT5 by TPO induces global re-localization to canonical phosphorylated STAT5 (pSTAT5) enhancers, activating the megakaryocytic differentiation program [142].

As the JAK-STAT pathway is crucial for growth and survival of immune cells, gain-of-function mutations in *JAKs* are mostly found in haematological malignancies. In myeloproliferative neoplasms, the *JAK2*^V617F^ mutation accounts for 98% of cases of polycythaemia vera and around 50% of essential thrombocythaemia and myelofibrosis [143,144,145,146,147]. Various mutations in *JAK1*, *JAK2* and *JAK3* have been found in lymphoblastic leukaemia [148,149,150]. *STAT3*, *STAT5* and *STAT6* also carry somatic mutations in B- and T-cell leukaemias [151,152]. *STAT3* overexpression has been noted in solid tumours as in uterine, lung, ovarian, gastric and brain cancers [153].

Missense and non-frameshift indel mutations usually affecting the pseudokinase domain of JAK1, JAK2 and JAK3 are frequently found in ML-DS and in some TMD cases [9,10,21,154,155,156,157,158,159,160,161,162]. Such mutations cause the constitutive activation of JAKs, since the JAK pseudokinase domain negatively regulates JAK activity [163]. The TPO receptor MPL is also commonly affected by missense mutations in ML-DS patients [9,21]. The overexpression of *Mpl*^W515L^ in bone marrow cells from *Gata1s*/Ts1Rhr mice causes thrombocytosis and intense bone marrow fibrosis, leading to lethal leukaemia in recipient mice [31]. In addition, a clonal variant A455D in *CSF2RB*, a coreceptor of IL-3, IL-5 and GM-CSF, was detected in 4.7% of ML-DS patients [21]. CSF2RB is involved in survival, proliferation and differentiation of haematopoietic cells as it interacts with cytokines to trigger JAK-STAT signalling [164,165]. Mechanistically, the A455D variant promotes ligand-independent activation of STAT5 through JAK and, when transduced in HSPCs, impairs terminal megakaryocytic differentiation and promotes the preferential growth of erythrocytes. Ruxolitinib, a FDA-approved JAK inhibitor (jakinib) for the treatment of myeloproliferative neoplasms, can reverse the abnormal expansion of erythroid progenitors [21]. There are other jakinibs in clinical trials and tofacitinib is also approved for the treatment of rheumatoid arthritis [166]. Further studies will elucidate whether jakinibs may be a viable therapeutic strategy for ML-DS with JAK-STAT mutations.

#### 4.2.2. Mutations in RAS Members

RAS proteins belong to a superfamily of low molecular weight GTP-binding proteins that are involved in signalling transduction and control of cell proliferation and survival. RAS proteins are active when they are bound to GTP and inactive when bound to GDP [167]. Activation occurs upon binding of specific ligands to receptor tyrosine kinases (RTKs). RTKs include important haematopoietic receptors such as KIT and FLT3. Active RAS triggers a phosphorylation cascade that ends up in the phosphorylation of ERK1/2 and subsequent activation of AP1 transcription factors to promote cell-cycle [168]. PI3K is another main effector activated by RAS, which results in a strong anti-apoptotic function and generation of survival signals [169,170].

Oncogenic mutations in *RAS* genes are present in 20% of all human tumours, most frequently in *KRAS* (85%), *NRAS* (15%) and *HRAS* (fewer than 1% of cases) [171]. The main mutations in *RAS* genes compromise the hydrolysis of GTP, and consequently RAS accumulates in the GTP-bound, active form. *KRAS* is predominantly mutated in solid tumours (100% of pancreatic ductal adenocarcinoma [172], 42% of colorectal adenocarcinoma [173] and 33% of lung adenocarcinoma cases [174]), whereas *NRAS* is the main mutated form in leukaemia (15%) [57]. The RAS-RAF-MEK-ERK signalling cascade plays a central role in the pathogenesis of AML, and genetic alterations in upstream activators or members of the pathway (not only RAS) are frequently observed in this disease. These alterations result in the constitutive phosphorylation of ERK in more than 50% of AML cases [175,176]. RAS-signalling mutations skew haematopoiesis to the myelomonocytic lineage and promote increased proliferation. In CMML, the presence of oncogenic RAS pathway genes has been associated with a more aggressive subtype and leukaemic transformation towards AML [177]. There are other pathway genes involved in leukaemia such as PTPN11 which is mutated in 4% of AML patients [57]. In MDS, mutations have been described in all three RAS genes, although at slightly lower frequencies than in AML [176,178]. In ML-DS, missense mutations affecting *KRAS*, *NRAS* and *NF1* are found in 14% of patients. The majority are gain-of-function as they involve the GTP-binding domain [9,21]. Paediatric non-DS AMKL cases also have mutations in *NRAS*, *KRAS* and *PTPN11* (15.7%) [55].

The development of drugs targeting mutant RAS proteins has yielded almost no molecules available for clinical use [179]. However, a recent exception has been the approval by the FDA of a small-molecule compound (sotorasib) specifically targeting KRAS^G12C^ in non-small cell lung cancer (NSCLC) [180]. An alternative strategy is to target downstream effectors such as ERK, RAF or MEK. Several RAS pathway inhibitors are being currently tested on clinical trials [181]. This could potentially be a suitable strategy for some ML-DS cases, as myeloid neoplasms with co-occurring mutations in *EZH2* show increased dependency on RAS signalling, which renders tumour cells more sensitive to MEK inhibitors [182].

## 5. Conclusions

Three main genetic elements characterize ML-DS: trisomy 21, mutations in *GATA1*, and secondary lesions in transcriptional regulators and signalling proteins. While the aberrant foetal haematopoietic differentiation of trisomy 21 may provide a basis for the selective advantage of clones with *GATA1* mutations, it is yet unclear why mutations in cohesin and *CTCF* are so abundant in ML-DS. In particular, several questions are still unanswered: How is the 3D genome organization affected by cohesin and *CTCF* mutations in ML-DS? What genes and pathways are deregulated? Do they cooperate or synergize with chromosome 21 genes or GATA1s? Do cohesin and *CTCF* mutations cause the same oncogenic effects, or alternatively, do they promote ML-DS through different mechanisms? Cellular and animal models that incorporate all the elements of this disease will be key in providing answers to these questions.

The well-defined sequence of events that leads to ML-DS represents a suitable model to understand the stepwise acquisition of mutations that is thought to occur during the clonal expansion leading to AML. Moreover, most mutations found in ML-DS are also found in AML, albeit at different frequencies. Current data indicate that the specific combination and order in which mutations are acquired is critical for AML development [92,183,184]. Therefore, mechanistic insights from the study of ML-DS may provide conceptual advances and practical benefits to patients with non-DS myeloid malignancies such as AML.

## Data Availability

Not applicable.
